# Supplementary material for: Breaking Entry-and Species Barriers: LentiBOOST® Plus Polybrene Enhances Transduction Efficacy of Dendritic Cells and Monocytes by Adenovirus 5
Source: Viruses. 2022 Jan 5;14(1):92. doi: 10.3390/v14010092 (PMC8781300; doi:10.3390/v14010092)
Supplement: Supplementary file 1 [file viruses-14-00092-s001.zip › viruses-1540482-supplementary.pdf]

Supplementary Information

Breaking entry- and species barriers: LentiBOOST® plus Polybrene enhances transduction efficacy of dendritic cells and monocytes by adenovirus 5

Astrid Strack[1,*], Andrea Deinzer[1,2], Christian Thirion[3], Silke Schrödel[3], Jan Dörrie[4], Tatjana Sauerer[4], Alexander Steinkasserer[1] & Ilka Knippertz[1,*]

Department of Immune Modulation, Universitätsklinikum Erlangen, Friedrich-Alexander Universität Erlangen-Nürnberg, Hartmannstr. 14, 91052 Erlangen, Germany; andrea.deinzer@uk-erlangen.de (A.D.); alexander.steinkasserer@uk-erlangen.de (A.S.)

[2] Institute of Clinical Microbiology, Immunology and Hygiene, Universitätsklinikum Erlangen,
Friedrich-Alexander Universität Erlangen-Nürnberg, Wasserturmstraße 3/5, 91054 Erlangen, Germany

[3] SIRION Biotech GmbH, Am Klopferspitz 19, 82152 Martinsried, Germany; Thirion@sirion-biotech.de (C.T.); Schroedel@sirion-biotech.de (S.S.)

[4] Department of Dermatology, Universitätsklinikum Erlangen, Friedrich-Alexander Universität
Erlangen-Nürnberg, Hartmannstr. 14, 91052 Erlangen, Germany; Jan.Doerrie@uk-erlangen.de (J.D.);
tatjana.sauerer@uk-erlangen.de (T.S.)

***** Correspondence: astrid.strack@uk-erlangen.de (A.S.); ilka.knippertz@uk-erlangen.de (I.K.)

Supplementary Information

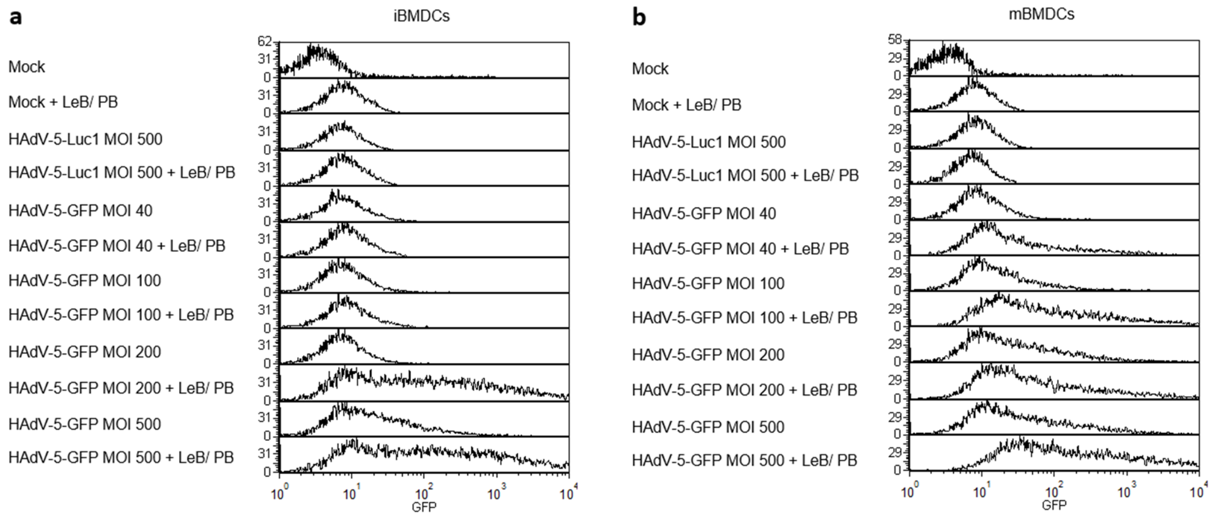

Supplementary Figure S1. Distribution of GFP+ BMDCs. Immature (i)BMDCs (a) and LPS-matured (m)BMDCs (b) were transduced with HAdV-5-GFP at different MOIs in combination with LentiBOOST®/Polybrene (+LeB/PB) or PBS only (-LeB/PB). As a negative control, cells were not transduced ("Mock") or transduced with HAdV-5-Luc1. Values were normalized to peak value. One representative experiment out of four is shown.

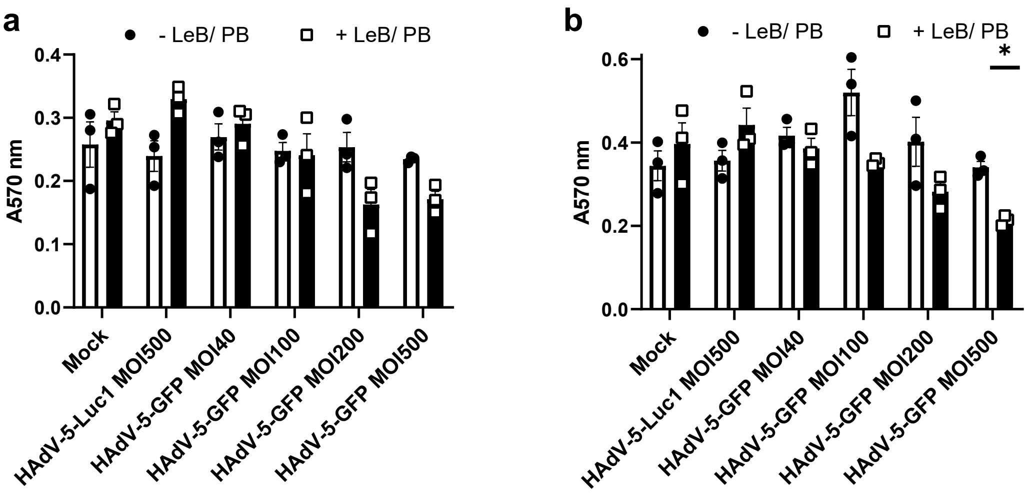

Supplementary Figure S2. BMDC viability assessed by an MTT assay. Immature (a) and mature (b) BMDCs were transduced at different MOIs together with LentiBOOST®/Polybrene (+LeB/PB) or PBS (-LeB/PB). Non-transduced ("Mock") or HAdV-5-Luc1 transduced cells served as controls. Twenty-thousand cells were cultivated with 100 µg MTT per well for 5 h. Absorbance at 570 nm was measured using a Wallac Victor 2 1420 Multilabel Counter (Perkin Elmer). Data are mean ± SEM of three different mice. Two-way ANOVA and Sidak correction were performed. * $P < 0.05$, bars without annotation are not significant ($P > 0.05$) in comparison to the respective condition –LeB/ PB.

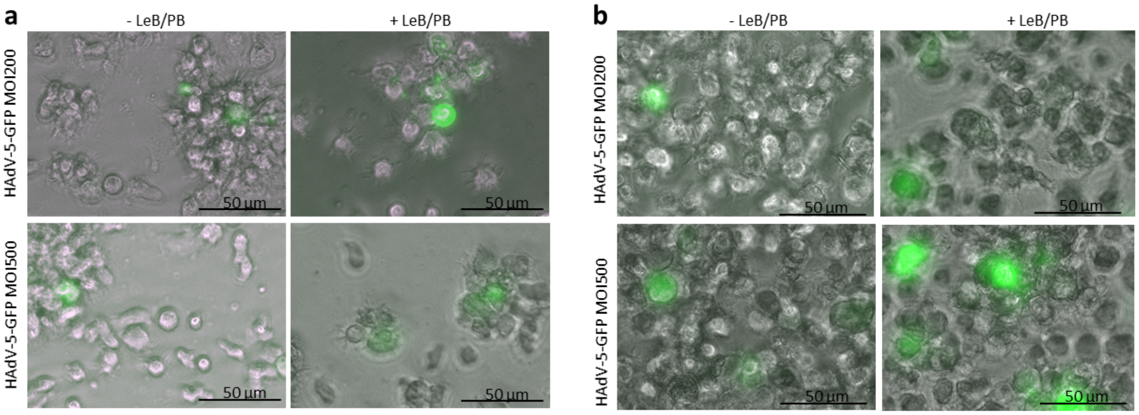

Supplementary Figure S3: Morphology of immature (a) and mature (b) murine BMDCs transduced with HAdV-5-GFP with LentiBOOST®/Polybrene +LeB/PB) or PBS (-LeB/PB). Images were taken using a Keyence BZ-X800 All-in-one Fluorescence Microscope.

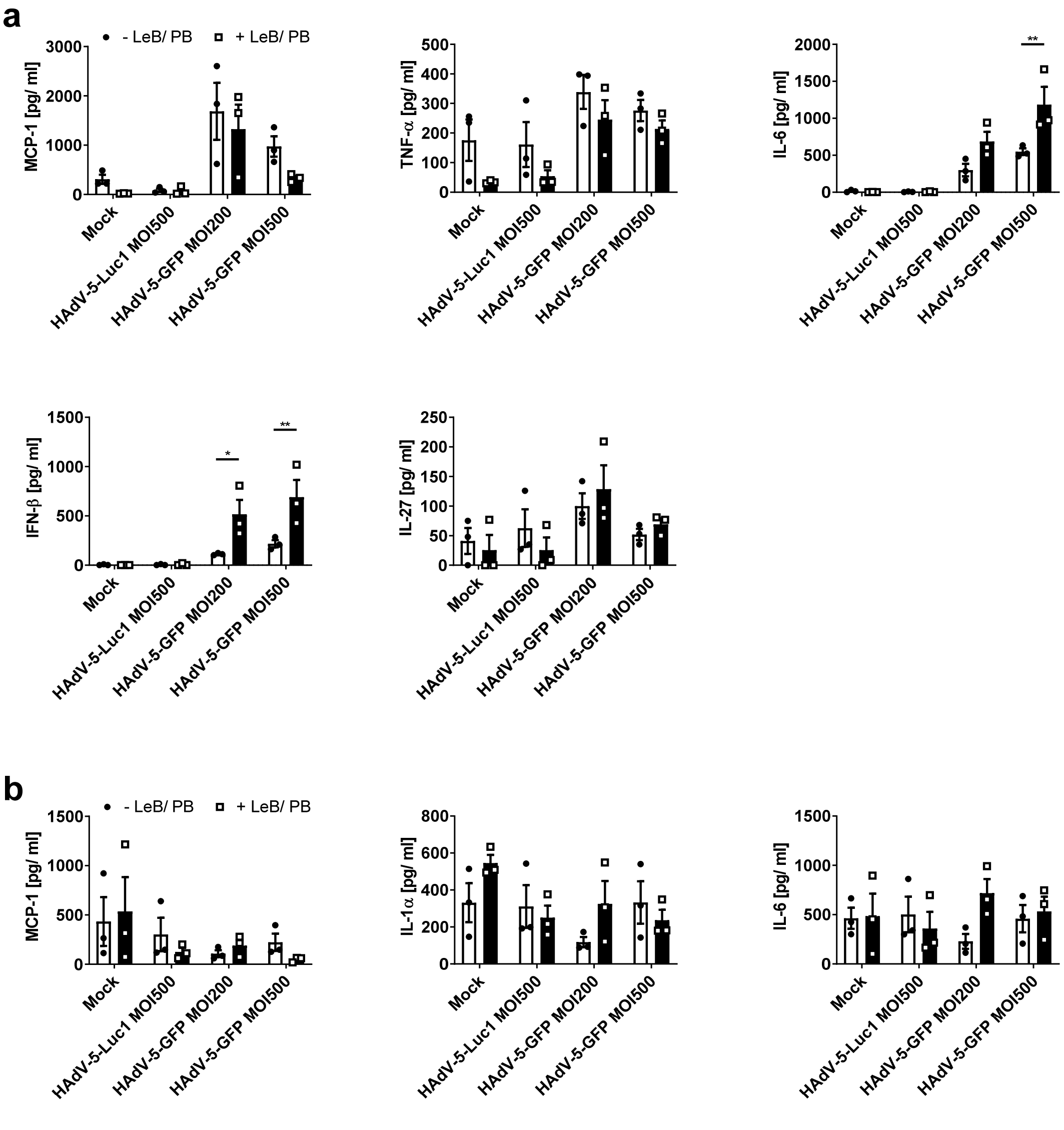

Supplementary Figure S4. LentiBOOST®/ Polybrene alters IL-6 and IFN-β secretion of immature BMDCs. Immature **(a)** and LPS-matured (m)BMDCs **(b)** were transduced with HAdV-5-GFP at different MOIs in combination with LentiBOOST®/ Polybrene (+LeB/ PB) or buffer only (-LeB/ PB), for 48 hours. As a control, cells were not transduced with a virus ("Mock") or using HAdV-5-Luc1. Cell culture supernatants from cells shown in figure 1 were analyzed for their content of cytokines using a cytometric bead array (CBA). Data are mean ± SEM of three independent experiments with cells derived from different mice. Two-way ANOVA and Tukey correction were performed. * $P < 0.05$, ** $P < 0.01$, bars without annotation are not significant ($P > 0.05$) in comparison to the respective condition –LeB/ PB.

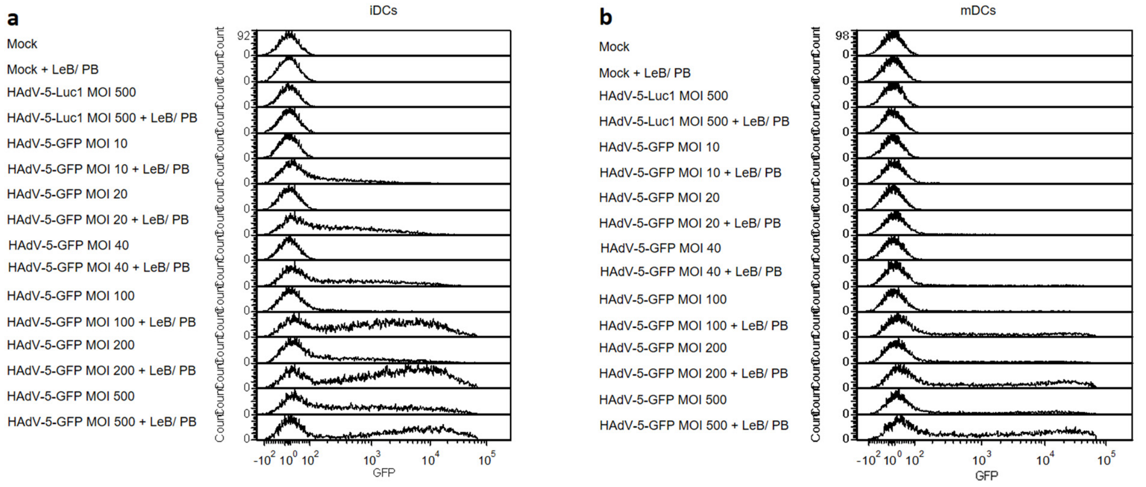

Supplementary Figure S5. Distribution of GFP+ human monocyte-derived DCs. Immature (i)DCs (a) and matured (m)DCs (b) were transduced with HAdV-5-GFP at different MOIs in combination with LentiBOOST®/Polybrene (+LeB/PB) or PBS only (-LeB/PB). Non-transduced ("Mock") or HAdV-5-Luc1-transduced cells served as negative controls. Values were normalized to peak value. One representative experiment out of three is shown.

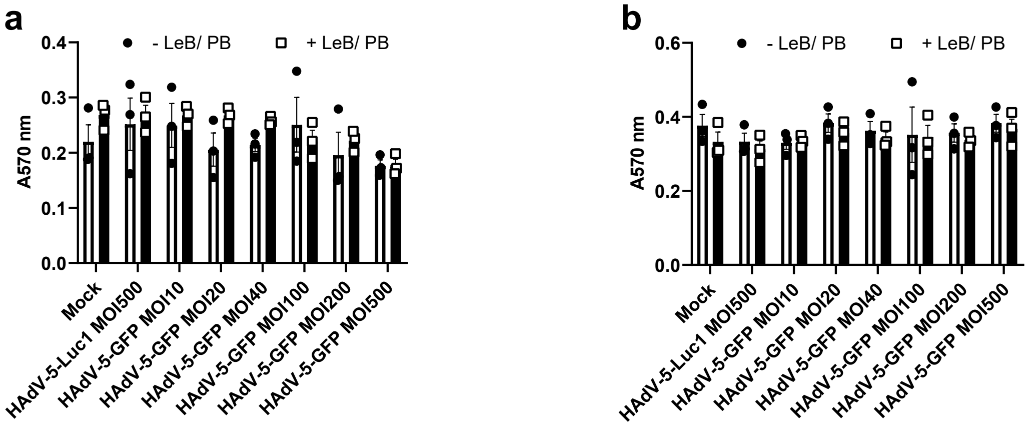

Supplementary Figure S6. Human DC viability assessed by an MTT assay. Immature (a) and mature (b) human monocyte-derived DCs were transduced at different MOIs in combination with LentiBOOST®/Polybrene (+LeB/PB) or PBS (-LeB/PB). Non-transduced ("Mock") or HAdV-5-Luc1 transduced cells served as controls. Per condition, 2×10^4 cells were cultivated with 100 µg MTT per well for 5 h. Absorbance at 570 nm was measured in duplicates using a Wallac Victor 2 1420 Multilabel Counter (Perkin Elmer). Data are mean ± SEM of three different mice. Two-way ANOVA and Sidak correction were performed. Bars without annotation are not significant ($P > 0.05$) in comparison to the respective condition –LeB/ PB.

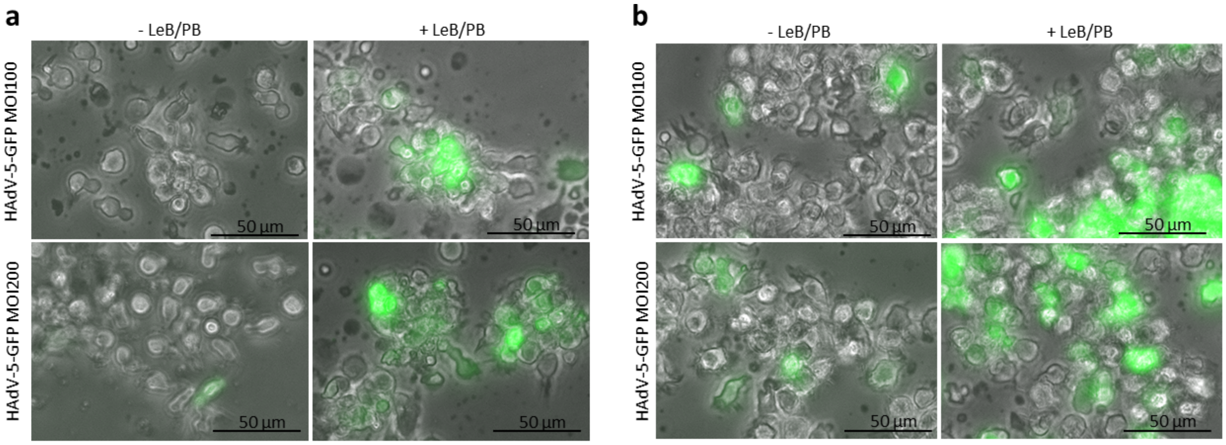

Supplementary Figure S7: Morphology of immature (a) and mature (b) human monocyte-derived DCs transduced with HAdV-5-GFP with LentiBOOST®/Polybrene +LeB/PB) or PBS (-LeB/PB). Images were taken using a Keyence BZ-X800 All-in-one Fluorescence Microscope.

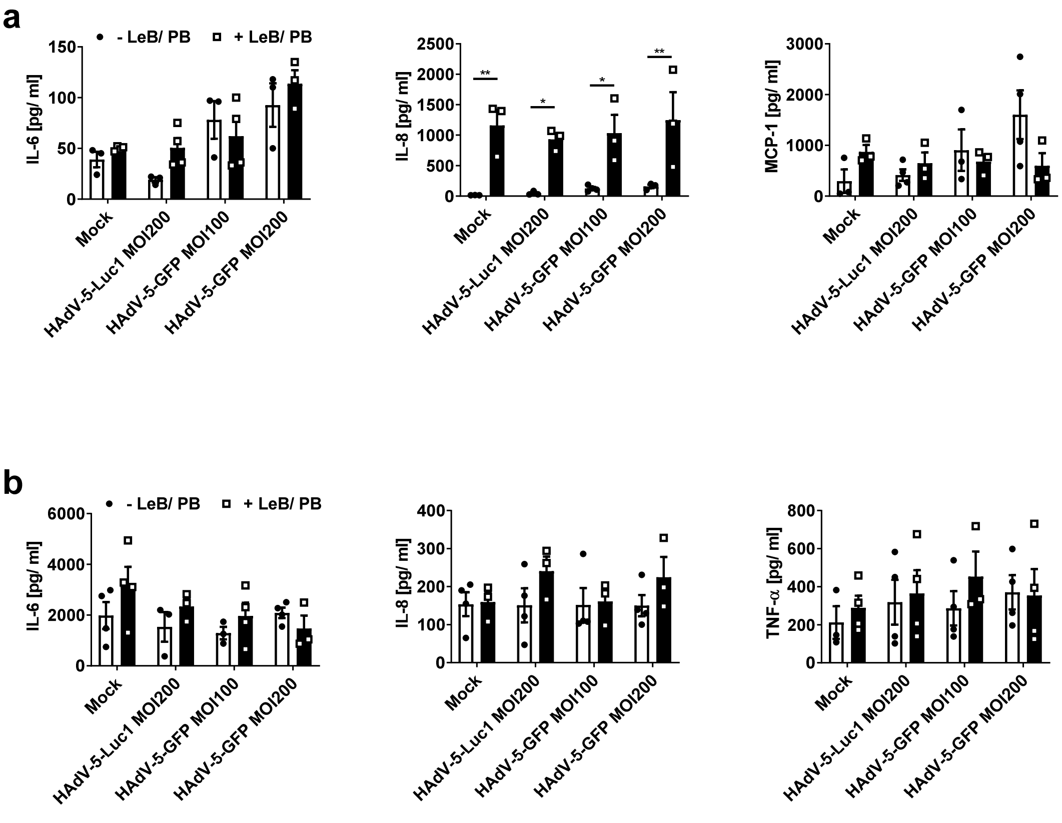

Supplementary Figure S8. LentiBOOST®/ Polybrene enhances IL-8 secretion by immature, but not by mature human DCs. Human monocyte-derived immature (i)DCs **(a)** and mature (m)DCs **(b)** were transduced with HAdV-5-Luc1 at a MOI of 200, or with HAdV-5-GFP at a MOI of 100 and 200 in combination with LentiBOOST®/ Polybrene (+LeB/ PB) or buffer only (-LeB/ PB), for 48 hours. Non-transduced cells ("Mock") served as a negative control. Cell culture supernatants from harvested DCs, shown in figure 5, were analyzed for secreted cytokines using CBA. Data are mean ± SEM of three independent experiments with cells derived from different healthy donors. Two-way ANOVA and Sidak correction were performed. * $P < 0.05$, ** $P < 0.01$, bars without annotation are not significant ($P > 0.05$) in comparison to the respective condition –LeB/ PB.

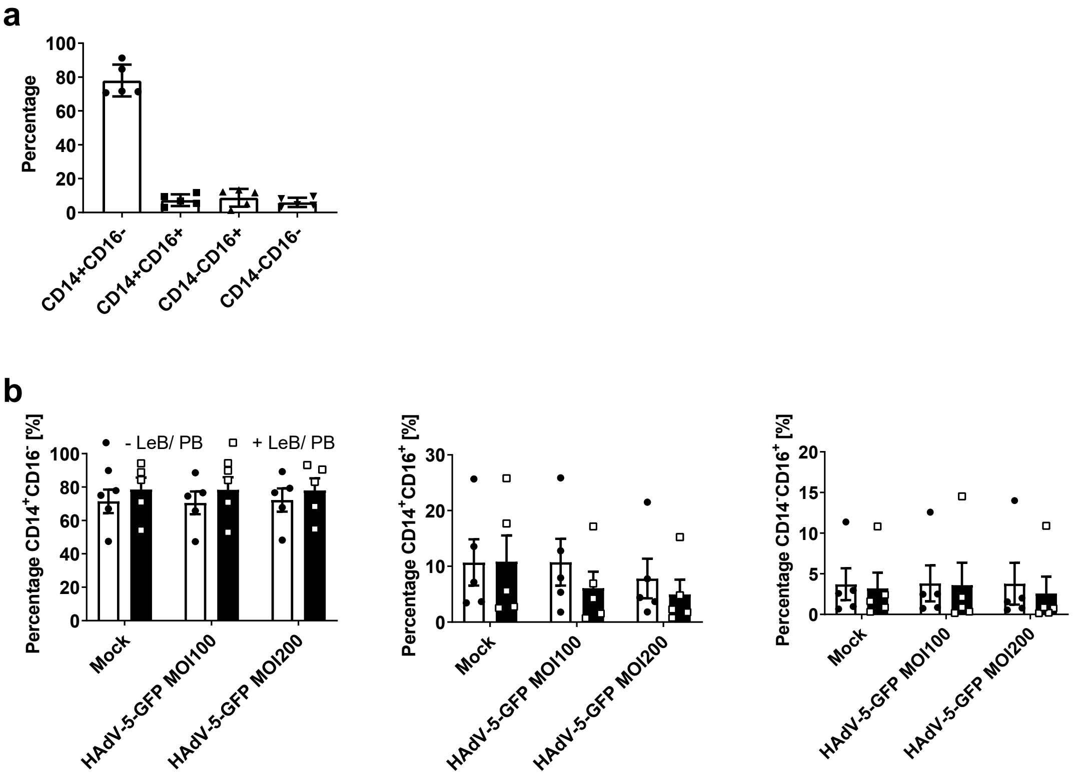

Supplementary Figure S9. Differentiation of human monocyte subsets from PBMCs is not influenced by adenoviral transduction facilitated by LentiBOOST®/ Polybrene. Human PBMCs isolated from leukocyte reduction system chambers were analyzed using flow cytometry before and after adenoviral transduction with HAdV-5-GFP. **(a)** Flow cytometric analyses of freshly isolated living 7-AAD negative, lineage negative HLA-DR+ monocytes, further subdivided into CD14+CD16- classical monocytes, CD14+CD16+ intermediate monocytes, CD14-CD16+ non-classical monocytes, and CD14-CD16- DCs. **(b)** Twenty-four hours post adenoviral transduction with an MOI of 100 or 200 in the presence of LentiBOOST®/ Polybrene (+LeB/ PB) or buffer only (- LeB/ PB), cells were analyzed using flow cytometry as described above. Non-transduced (“Mock”) cells served as a control. Data are mean ± SEM for five different experiments. Two-way ANOVA and Sidak correction were performed. Bars without annotation are not significant ($P > 0.05$) in comparison to the respective condition without LeB/ PB.

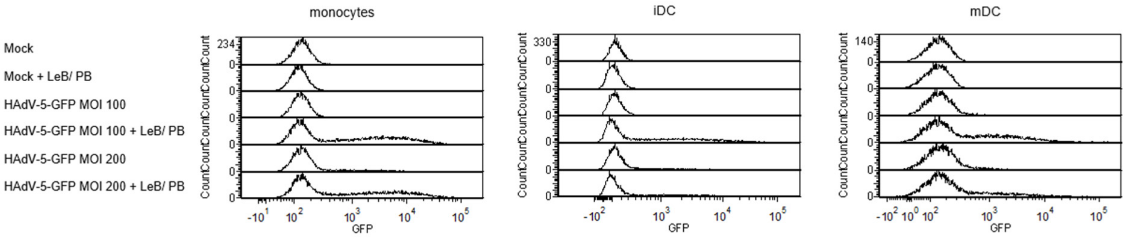

Supplementary Figure S10. Distribution of GFP⁺ human monocytes and monocyte-derived DCs. Human monocytes were transduced with HAdV-5-GFP at different MOIs in combination with LentiBOOST®/Polybrene (+LeB/PB) or PBS only (-LeB/PB) (left panel) and subsequently differentiated into immature (middle panel) and mature (right panel) DCs. Non-transduced (“Mock”) or HAdV-5-Luc1-transduced cells served as negative controls. Depicted is the distribution of GFP signal normalized to the peak value. One representative experiment out of fiveis shown.

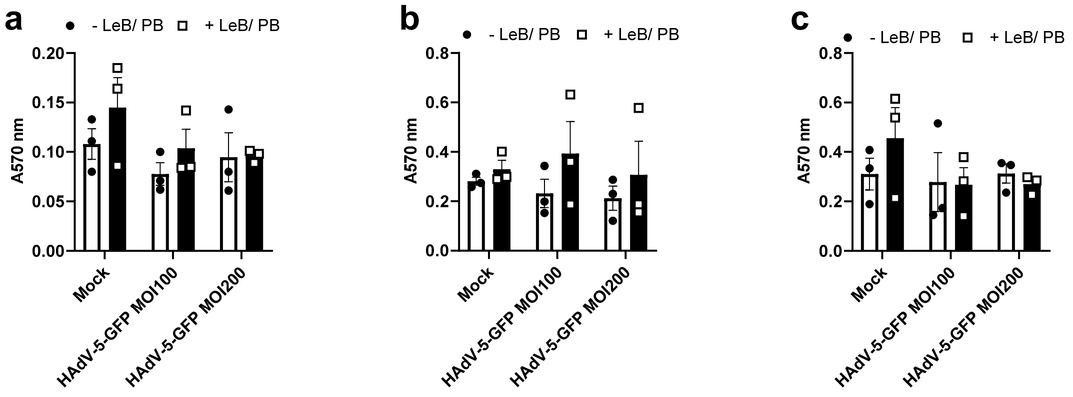

Supplementary Figure S11. Viability of Monocytes assessed by a MTT assay. Human monocytes (a) were transduced at different MOIs in combination with LentiBOOST®/Polybrene (+LeB/PB) or PBS (-LeB/PB). Non-transduced ("Mock") or HAdV-5-Luc1 transduced cells served as controls. Transduced monocytes were further differentiated into immature (b) and mature (c) DCs. Per condition, $2x10^4$ cells were cultivated with 100 µg MTT per well for 5 h. Absorbance at 570 nm was measured in duplicates using a Wallac Victor 2 1420 Multilabel Counter (Perkin Elmer). Data are mean ± SEM of three different mice. Two-way ANOVA and Sidak correction were performed. Bars without annotation are not significant (P > 0.05) in comparison to the respective condition –LeB/ PB.

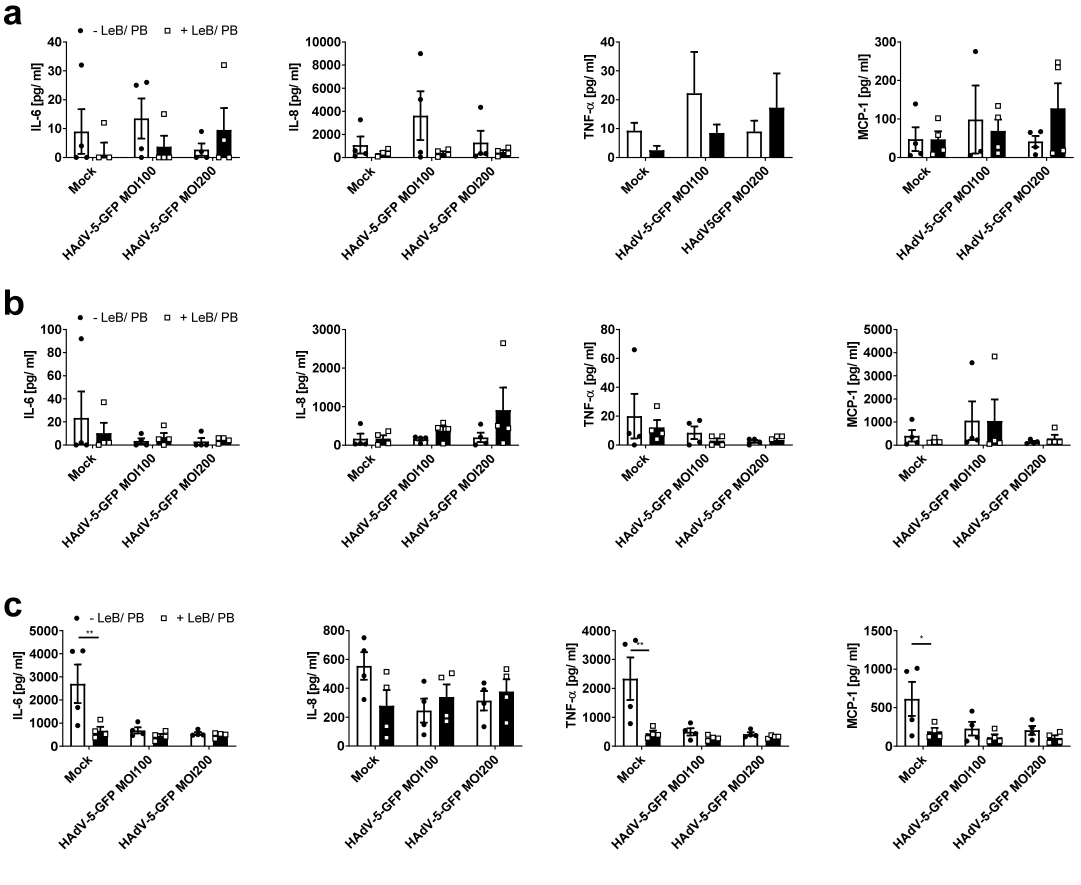

Supplementary Figure S12. LentiBOOST®/ Polybrene alters secretion of pro-inflammatory cytokines in untransduced mDCs. Monocytes were not transduced ("Mock") or transduced with HAdV-5-GFP (MOI100 and MOI200) using LentiBOOST®/ Polybrene (+LeB/ PB) or buffer only (-LeB/ PB). Afterwards differentiation of monocytes into iDCs was induced by adding GM-CSF and IL-4 for 4 days to the cell culture. Addition of IL-1β, IL-6, TNF α, and PGE2 for another 24 hours resulted in mDCs. **(a-c)** Cytometric bead array (CBA) of cell culture supernatants to determine the cytokine content derived from monocytes 24 hours post infection **(a)**, iDCs at day 4 **(b)** and mDCs at day 5 **(c)**, shown in figure 7. Data are mean ± SEM of three different experiments. Two-way ANOVA and Sidak correction were performed. * $P < 0.05$, ** $P < 0.01$, bars without annotation are not significant ($P > 0.05$) in comparison to the respective condition –LeB/ PB.

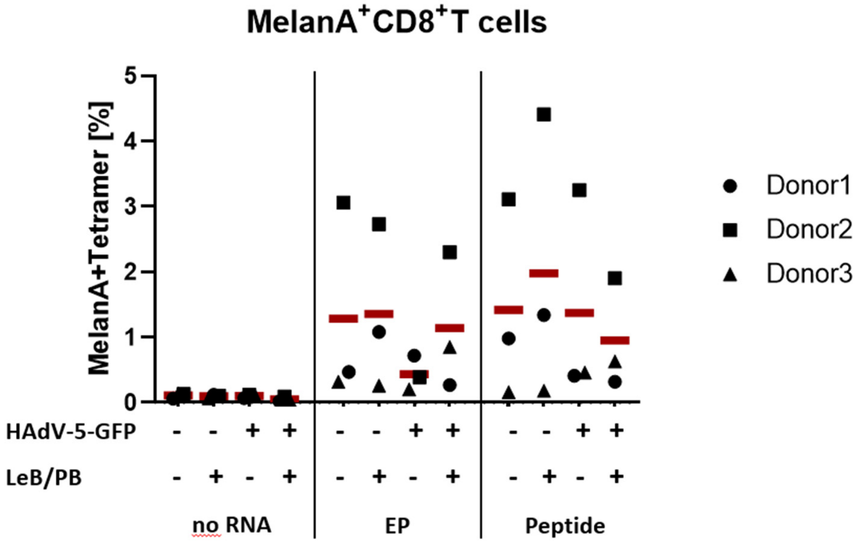

Supplementary Figure S13. Mature DCs derived from transduced monocytes retain their capability to prime autologous T cells in a tumor-antigen-specific manner. DCs treated as described in figure 8, were used to stimulate autologous CD8+ T cells at a 1:10 ratio. One week after stimulation, the percentage of MelanA-specific T cells was analyzed by tetramer-staining and flow-cytometry. The indicated percentage is calculated in reference to all CD8+ T cells. Data are mean of three independent experiments with cells derived from different healthy HLA A2+ donors. Two-way ANOVA and Sidak correction were performed. Bars without annotation are not significant ($P > 0.05$) in comparison to the respective condition –LeB/ PB.
